# LECT2 as a hepatokine links liver steatosis to inflammation via activating tissue macrophages in NASH

**DOI:** 10.1038/s41598-020-80689-0

**Published:** 2021-01-12

**Authors:** Noboru Takata, Kiyo-aki Ishii, Hiroaki Takayama, Mayumi Nagashimada, Kyoko Kamoshita, Takeo Tanaka, Akihiro Kikuchi, Yumie Takeshita, Yukako Matsumoto, Tsuguhito Ota, Yasuhiko Yamamoto, Satoshi Yamagoe, Akihiro Seki, Yoshio Sakai, Shuichi Kaneko, Toshinari Takamura

**Affiliations:** 1 Department of Endocrinology and Metabolism, Kanazawa University Graduate School of Medical Sciences, Kanazawa, Ishikawa 920-8640 Japan; 2 Department of Gastroenterology, Kanazawa University Graduate School of Medical Sciences, Kanazawa, Ishikawa 920-8640 Japan; 3 Technology Department of Clinical Laboratory Science, Kanazawa University Graduate School of Medical Science and Technology, Kanazawa, Ishikawa 920-0942 Japan; 4 Department of Biochemistry and Molecular Vascular Biology, Kanazawa University Graduate School of Medical Science, Kanazawa, Ishikawa 920-8640 Japan; 5 Department of Chemotherapy and Mycoses, National Institute of Infectious Diseases, Shinjuku-ku, Tokyo, 162-8640 Japan; 6 Life Sciences Division, Engineering and Technology Department, Kanazawa University, Kanazawa, Ishikawa 920-8640 Japan; 7 Department of Integrative Medicine for Longevity, Kanazawa University Graduate School of Medical Sciences, Kanazawa, Ishikawa 920-8640 Japan

**Keywords:** Obesity, Non-alcoholic fatty liver disease, Non-alcoholic steatohepatitis

## Abstract

It remains unclear how hepatic steatosis links to inflammation. Leukocyte cell-derived chemotaxin 2 (LECT2) is a hepatokine that senses fat in the liver and is upregulated prior to weight gain. The aim of this study was to investigate the significance of LECT2 in the development of nonalcoholic steatohepatitis (NASH). In human liver biopsy samples, elevated *LECT2* mRNA levels were positively correlated with body mass index (BMI) and increased in patients who have steatosis and inflammation in the liver. *LECT2* mRNA levels were also positively correlated with the mRNA levels of the inflammatory genes *CCR2* and *TLR4*. In C57BL/6J mice fed with a high-fat diet, mRNA levels of the inflammatory cytokines *Tnfa* and *Nos2* were significantly lower in *Lect2* KO mice. In flow cytometry analyses, the number of M1-like macrophages and M1/M2 ratio were significantly lower in *Lect2* KO mice than in WT mice. In KUP5, mouse kupffer cell line**,** LECT2 selectively enhanced the LPS-induced phosphorylation of JNK, but not that of ERK and p38. Consistently, LECT2 enhanced the LPS-induced phosphorylation of MKK4 and TAB2, upstream activators of JNK. Hepatic expression of LECT2 is upregulated in association with the inflammatory signature in human liver tissues. The elevation of LECT2 shifts liver residual macrophage to the M1-like phenotype, and contributes to the development of liver inflammation. These findings shed light on the hepatokine LECT2 as a potential therapeutic target that can dissociate liver steatosis from inflammation.

## Introduction

Nonalcoholic fatty liver disease (NAFLD) is one of the most common forms of chronic liver disease and is closely related to overnutrition, metabolic syndrome, and insulin resistance^1^. Nonalcoholic steatohepatitis (NASH), which develops in approximately 20% of NAFLD patients, is characterized by hepatocellular injury followed by the development of inflammation and fibrosis. Recent studies suggest that several diverse processes occurring in parallel contribute to the development of liver inflammation^2^. However, the causal factors that connect overnutrition to liver inflammation are not fully elucidated.

The liver is a major site for the production of bioactive secretory proteins called hepatokines. Several lines of evidence suggest that the dysregulated production of hepatokines, such as selenoprotein P or fetuin-A, are involved in the development of insulin resistance^3,4^. The leukocyte cell-derived chemotaxin 2 (LECT2) was initially identified as a chemotactic factor for neutrophils^5^. We rediscovered it as an obesity-associated hepatokine. Hepatic expression of *LECT2* is closely associated with body mass index (BMI) in humans and is negatively regulated by the energy depletion-sensing protein adenosine monophosphate-activated protein kinase (AMPK)^6^. Both LECT2 gene expression in the liver and LECT2 protein levels in the blood are downregulated in mice that have been fasted for 12 h^6^. In contrast, a high-fat intake for more than 1 week increased *LECT2* gene expression in the liver and LECT2 protein levels in the blood of mice^6^. Specifically, circulating LECT2 levels in the blood reflects the content of hepatic triglycerides rather than body fat, and predicts the onset of weight cycling in mice^7^. These findings indicate that LECT2 is an energy-sensing hepatokine that is upregulated in response to overnutrition. To date, LECT2 is known to be involved in many pathological conditions, such as sepsis^8^, diabetes^6^, systemic amyloidosis^9^, hepatocarcinogenesis^10^, NAFLD^11^, and liver fibrosis^12^. However, the role of LECT2 in the development of liver inflammation, which can bridge the gap from steatosis to fibrosis in the development of NASH, remains largely unclear.

It has been reported that LECT2 is a modulator of both immune and inflammatory reactions. Lu et al. showed that treatment with LECT2 improves protective immunity by enhancing macrophage functions in septic mice^8^. Hwang et al. reported that LECT2 induces an atherosclerotic inflammatory reaction via CD209 receptor-mediated c-Jun N-terminal kinase (JNK) phosphorylation in human endothelial cells^13^. Based on these findings, we hypothesized that LECT2 links overnutrition to the development of liver inflammation and the subsequent development of NASH. In the present study, we tested this hypothesis by investigating the significance of LECT2 gene expression in human liver tissues and the role of LECT2 in liver inflammation and the subsequent development of NASH in a mouse model of inflammation.

## Results

### Hepatic *LECT2* expressions positively correlate with liver inflammation and steatosis

The initial findings of a possible connection between LECT2 and liver inflammation came from our genetic analyses of liver tissue samples from human subjects^14,15^. The cohort consisted of 32 people with a wide range of BMIs with mild liver steatosis. The clinical backgrounds of the study subjects are shown in Table 1.

**Table 1:** Clinical characteristics of the study subjects.

n	32
Gender (M/F)	21/11
Age (years)	51 (28–70)
Body mass index (kg/m^2^)	24.5 (19.4–34.9)
White blood cell (/µL)	5450 (3200–9700)
Hemoglobin (g/dL)	14.1 (10.0–17.1)
Platelet (× 10^4^/µL)	21.1 (14.8–41.2)
Fasting plasma glucose (mg/dL)	110 (76–285)
Hemoglobin A1c (%)	6.4 (4.6–10.4)
HOMA-IR	2.49 (0.8–5.2)
Total bilirubin (mg/dL)	0.8 (0.4–1.5)
AST (IU/L)	22 (9–66)
ALT (IU/L)	26 (5–218)
ALP (IU/L)	229 (93–425)
Gamma-glutamyltransferase (IU/L)	52 (13–554)
Triglyceride (mg/dL)	106 (36–378)
Total cholesterol (mg/dL)	203 (113–292)
High-density lipoprotein cholesterol (mg/dL)	45 (28–84)
**Liver histological findings**	
Fibrosis (0/1/2/3/4)	20/12/0/0
Steatosis (0/1/2/3)	4/13/7/8
Lobular inflammation (0/1/2/3)	17/12/1/1

*HOMA-IR* Homeostasis Model Assessment of Insulin Resistance, *AST* aspartate aminotransferase, *ALT* alanine aminotransferase, *ALP* alkaline phosphatase.

**Figure 1:**
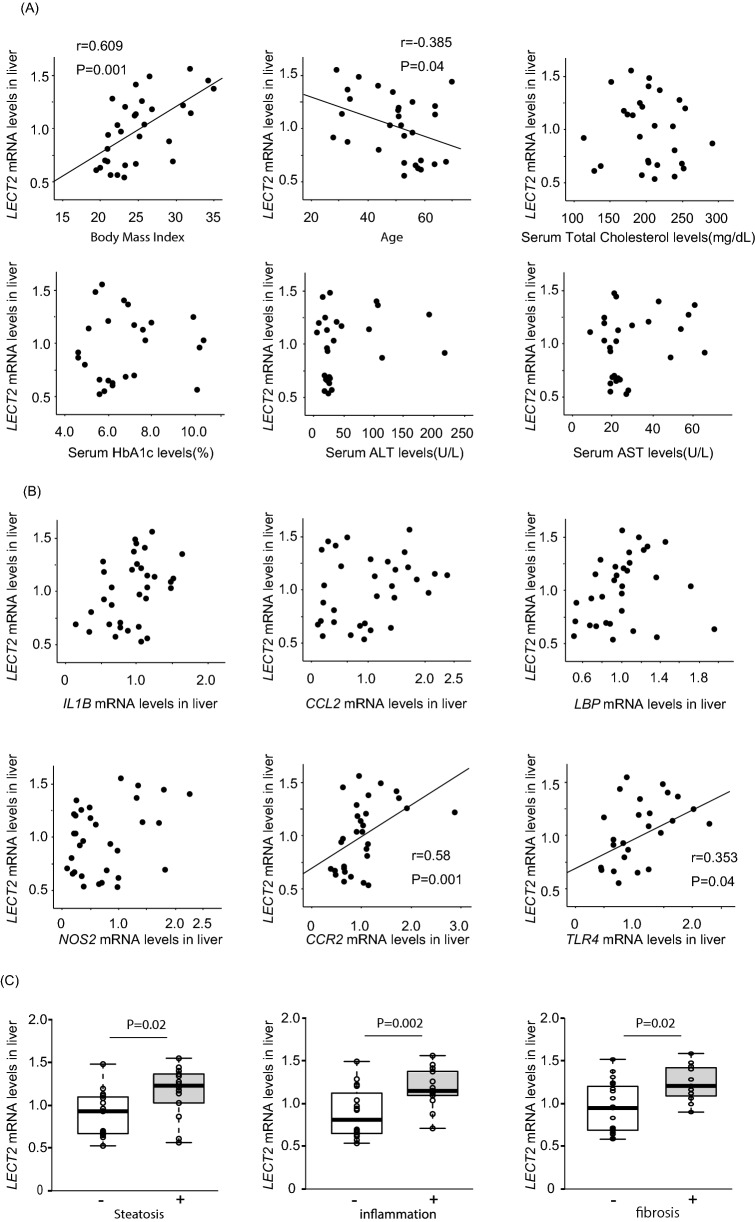
Hepatic *LECT2* gene expression level correlates with liver steatosis and gene expression levels of inflammation-related genes in the liver. (**A**) Correlations between *LECT2* gene expression in the liver and BMI, age, T-Chol, FPG, HbA1c, ALT, AST in humans (n = 32). (**B**) Correlations between *LECT2* gene expression in the liver and *IL-1b*, *CCL2*, *LBP*, *NOS2*, *CCR2*, *TLR4* in humans (n = 32). (**C**) Correlations between *LECT2* gene expression in the liver and Pathological inflammation and steatosis in humans (n = 32). *P < 0.05.

We performed liver biopsies and conducted a comprehensive analysis of the gene expression profiles. As we previously reported, *LECT2* mRNA levels were positively correlated with BMI (r = 0.609, P = 0.001), but not with serum levels of aspartate transferase (AST), alanine transaminase (ALT), and hemoglobin A1c (HbA1c) (Fig. 1A).

In liver histological findings, *LECT2* mRNA expression was higher in patients who have greater than grade1 steatosis than in those who have not (P = 0.02) (Fig. 1C). *LECT2* mRNA expression was also higher in patients with greater than grade 1 inflammation and greater than stage 1 fibrosis than those who have not (P = 0.002, 0.02, respectively) (Fig. 1C).

*LECT2* mRNA expression levels were also positively correlated with the mRNA expression levels of the inflammatory genes, *CCR2* (r = 0.457, P = 0.009) and *TLR4* (r = 0.573, P = 0.001) (Fig. 1B). These results indicate that *LECT2* mRNA expression is positively associated with obesity and liver inflammation in humans.

### *Lect2* deficient mice ameliorate HFD-induced liver inflammation

**Figure 2:**
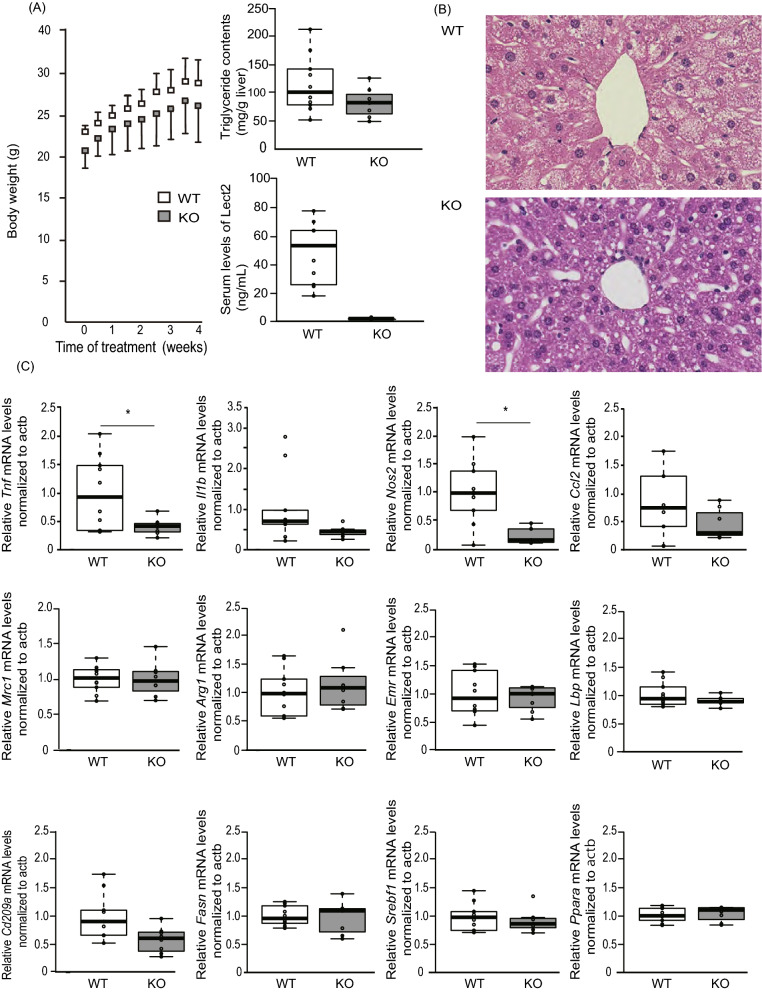
Lect2 KO mice attenuate diet-induced inflammation in the liver. (**A**) Graph shows bodyweight of Lect2 KO and wild-type mice fed an HFHS diet. 8 week-old-male mice were fed an HF diet for 4 weeks (n = 7–10). The graph shows the liver triglyceride content of Lect2 KO and wild-type mice fed an HF diet. 8 week-old-male mice were fed an HF diet for 4 weeks (n = 7–10). Blood levels of LECT2 from Lect2 KO and wild-type mice following fasting for 12 h. 8 week-old-male mice were fed HF diet for 4 weeks. (**B**) Hematoxylin and eosin staining of liver from Lect2 KO and wild-type mice fed an HF diet. 8 week-old-male mice were fed an HF diet for 4 weeks (n = 7–10). (**C**) Graphs show mRNA levels of genes involved in inflammation and lipid metabolism in the liver of Lect2 KO and wild-type mice fed an HF diet. 8 week-old-male mice were fed an HF diet for 4 weeks. *P < 0.05.

To investigate the potential link between LECT2 and liver inflammation, we fed wild-type and *Lect2* KO C57BL6J mice a high-fat diet (HFD) for 4 weeks and then evaluated the inflammatory responses in the liver. Previously, we found that *Lect2* mRNA expression in the liver was overwhelmingly dominant compared with that in other tissues in mice^5^. Therefore we used systemic *Lect2* KO mice in the following experiments, although the animal models of liver-specific downregulation for *Lect2* might be more suitable. HFD increased body weight in a time-dependent manner (Fig. 2A). We previously observed that *Lect 2* KO mice are protected from developing obesity in HFD feeding for as long as 8 weeks^6^. In the present study, there were no significant differences in the development of hepatic steatosis and body weight between the wild-type and *Lect2* KO mice (Fig. 2A,B). Also, the liver weight to body weight ratios and visceral fat weight to body weight ratios were not different between the two genotypes (data not shown). Therefore, we were able to investigate the HFD-induced inflammatory responses without the influences of hepatic steatosis and weight gain. The mRNA levels of two genes involved in the regulation of inflammation, *Tnfa* and *Nos2*, were significantly lower in *Lect2* KO mice compared with wild-type mice (P = 0.001 and 0.01, respectively) (Fig. 2C). Conversely, the mRNA levels of genes involved in fatty acid synthesis (*Fas* and *Srebp1c)* and β-oxidation (*Ppara*) were not different between the two genotypes (Fig. 2C).

### Hepatic M1-like macrophages were decreased in *Lect2* deficient mice

**Figure 3:**
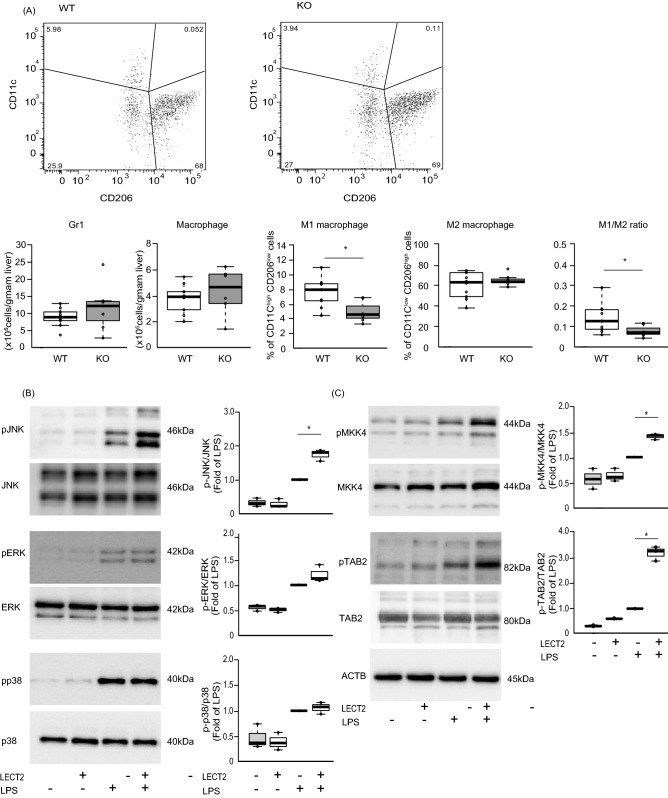
LECT2 changed the percentage of M1-like macrophages and M2-like macrophages in the liver of C57BL6 mice and enhanced LPS signaling in KUP5. (**A**) Flow cytometry analyses of the liver from wild-type and *Lect2* KO mice fed an HFD for 4 weeks. The representative plot demonstrates macrophage populations in the liver of *Lect2* KO mice fed an HFD. CD11c^high^/CD206^low^ and CD11c^low^ CD206^high^ populations were defined as M1-like and M2-like macrophages, respectively. Graphs show quantitation of Gr1-positive macrophages, F4/80 CD11b-positive macrophages, percentages of M1-like macrophages, and percentages of M2-like macrophages (n = 7, 10). B: Western blot analysis of LPS (400 ng/mL)-stimulated JNK, ERK and p38 phosphorylation in KUP5 treated with LECT2 (400 ng/mL). The cells were treated with LPS and LECT2 protein for 30 min (n = 3). (**C**) Western blot analysis of LPS (400 ng/mL)-stimulated MKK4 and TAB2 phosphorylation in KUP5 treated with LECT2 (400 ng/mL). The cells were treated with LPS and LECT2 protein for 30 min (n = 3). *P < 0.05.

The reduction in liver inflammation as a result of *Lect2* deficiency prompted us to investigate the subsets of macrophages. To quantify M1- and M2-like macrophages, we performed flow cytometry on the stromal vascular cells (SVCs) that were isolated from liver tissues of wild-type and *Lect2* KO mice fed a HFD for 4 weeks. Macrophages were identified as propidium iodide (PI)− CD45+ NK1.1− CD3− CD19− TER119− CD11b+ F4/80+ cells. There were no significant differences in the number of cells expressing Gr-1 and macrophages between wild-type and *Lect2* KO mice (Fig. 3A). CD11c^high^/CD206^low^ and CD11c^low^ CD206^high^ populations were defined as M1-like and M2-like macrophages, respectively. In flow cytometry analyses, the number of M1-like macrophages and M1/M2 ratio were significantly lower in *Lect2* KO mice than in wild-type mice (P = 0.006, P = 0.04, respectively) (Fig. 3A). Additionally, there were no significant differences in the number of M2-like macrophages between wild-type and *Lect2* KO mice.

In order to elucidate LECT2 effect in liver residual macrophage, we used KUP5, an immortalized mouse Kupffer cell line established from C57BL/6 strain^16^. We incubated KUP5 cells with recombinant LECT2 protein and lipopolysaccharide (LPS) for 30 min and checked mitogen-activated protein kinases (MAPKs) including c-jun N-terminal kinase (JNK), extracellular signal-regulated kinases (ERKs), and p38. LPS elevated the phosphorylation of all these MAPK family members, whereas LECT2 alone did not affect the phosphorylation of them. LECT2 selectively enhanced the LPS-induced phosphorylation of JNK, but not that of ERK and p38 (Fig. 3B). Indeed, LECT2 enhanced the LPS-induced phosphorylation of MKK4 and TAB2, upstream activators of JNK (Fig. 3C).

## Discussion

**Figure 4:**
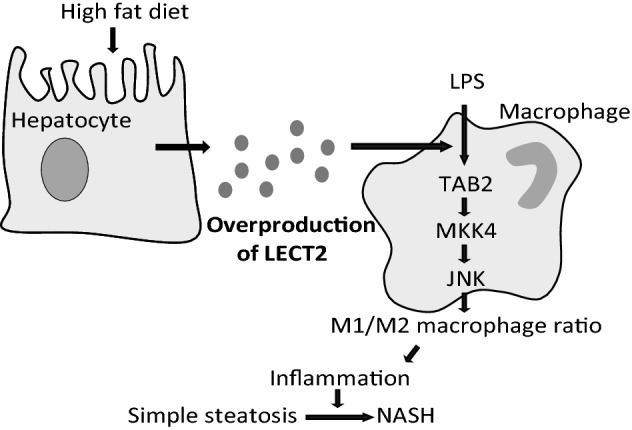
Proposed schema for the effects of LECT2 on liver inflammation. High-fat intake increased LECT2 protein levels in the blood and enhances LPS stimulated JNK phosphorylation which shifts liver macrophage to M1-like phenotype and causes inflammation in the liver.

In the present study, we demonstrated that LECT2 is a hepatokine that links obesity to hepatic inflammation via activation of LPS signaling in macrophages (Fig. 4). To the best of our knowledge, this is the first report to demonstrate that LECT2 plays a role in the development of liver inflammation and the progression from simple steatosis to NASH in the liver.

Previously, a two-hit model was proposed for the pathogenesis of NASH; a metabolic syndrome involving liver fat accumulation is the first hit, and the second hit is defined as the progression of fat accumulation to liver inflammation^17^. Alternatively, a multiple parallel hit model, which is a relatively new concept, has been proposed to explain the development of NASH; namely, many hits act in parallel, and inflammation may precede, rather than follow, steatosis^2^. Our previous study demonstrated that LECT2 is a hepatokine that senses overnutrition and liver fat accumulation and is upregulated prior to weight gain^7^. Based on the previous and current findings, we propose that hepatic steatosis and inflammation progress simultaneously during NASH development because liver fat accumulation induces LECT2, which drives inflammation.

To date, it has been reported that LECT2 also contributes to inflammation in many organs. Hwang et al. reported that LECT2 induces atherosclerotic inflammatory reaction in human endothelial cells^13^. Lu et al. have reported LECT2 treatment improves protective immunity by enhancing macrophage functions in septic mice^8^. However, the evidence for these inflammatory adaptations in humans remains lacking. In the present study, hepatic expressions of the *LECT2* gene was upregulated in tissue samples from patients with hepatic steatosis and inflammation, and both steatosis and inflammation were positively correlated with LECT2 gene expression. LECT2 upregulation may be causal for the subsequent inflammation in humans because *Lect2* deficient mice showed downregulated expression of inflammatory genes, such as *Tnf* and *Nos2*, in the liver. Even with short term HFD feeding, where body weight and liver steatosis were comparable between wild-type and *Lect2* deficient mice, *Lect2* deficient mice were protected from the M1-like shift of macrophages and induction of inflammatory genes in the liver.

Tosello et al. reported that macrophages have a crucial role in the early phase of NASH by producing inflammatory cytokines^18^. Tissue macrophages are phenotypically heterogeneous and are characterized according to their activation status as either 'classically activated' pro-inflammatory macrophages or 'alternatively activated' non-inflammatory macrophages. In the present study, *Lect2* deficiency did not change total macrophage number in the liver but reduced M1-like (F4/80+ CD11c+) macrophage number and M1/M2 (F4/80+ CD11c−) ratio of macrophages. Lumeng et al. showed that diet-induced obesity leads to a shift in the activation state of adipose tissue macrophages from an M2-like polarized state to an M1-like pro-inflammatory state, which contributes to insulin resistance^19^. Itoh et al. reported that a unique histological feature termed the hepatic crown-like structure, which consists of CD11c positive macrophages, is involved in the development of hepatic inflammation and fibrosis, and contributes to the disease progression from simple steatosis to NASH^20^. Based on these reports, together with our findings, we conclude that the overproduction of LECT2 in the liver may contribute to the M1-like macrophage-dominant phenotype and the subsequent liver inflammation.

JNK is a stress-sensing kinase that is activated by a variety of stimuli, including cytokines, reactive oxygen species, endoplasmic reticulum stress, and metabolic stresses^21^. Specifically, JNK is phosphorylated and activated by endoplasmic reticulum stress^22^ and saturated fatty acids^23^ in cultured hepatocytes as well as by HFD feeding in the liver of mice^21,24^. JNK activation has a significant role in the development of insulin resistance by inducing the phosphorylation of insulin receptor substrates at specific serine and threonine residues^25^. We found that *Lect2* deficient mice were protected from HFD-induced JNK phosphorylation and insulin resistance in the skeletal muscle^6^. In adipose tissue-infiltrating macrophages, JNK shifts pro-inflammatory M1-like macrophage polarization and thereby causes insulin resistance^26^. Based on these findings, we hypothesized that the JNK activation may be responsible for the LECT2-induced shift to M1-like macrophages.

Gut microbiota-derived LPS localizes in the liver of human and experimental NAFLD^27^ and may induce liver inflammation leading to the development of NASH^28^. Therefore, we used LPS as one of factors which induces phosphorylation of JNK and enhances inflammation in the liver macrophages. In the present study, LPS activates all of the MAPK family members, such as JNK, ERK, and p38. Treatment with recombinant LECT2 protein specifically enhanced the LPS-induced phosphorylation of JNK, but not that of ERK and p38, in the KUP5 kupffer cells. Consistently, LECT2 enhanced the LPS-induced phosphorylation of MKK4 and TAB2, upstream activators of JNK. These findings suggest that LECT2 specifically enhances the LPS-induced activation of the TAB2-MKK4-JNK axis in the MAPK signaling pathways as an adjuvant.

To date, a specific receptor for LECT2 mediated inflammation has not yet been identified. Unlike the findings in the present study, recombinant LECT2 alone is sufficient to activate JNK and thereby impair insulin signal transduction in C2C12 myotubes^6^. Also in human endothelial cells, LECT2 directly activates JNK and thereby induces inflammatory cytokines^13^. Identifying tissue-specific LECT2 receptors and its downstream signaling will help to further clarify the specific pathways leading to inflammation.

In addition, further investigation is needed as to why LECT2 specifically modifies the TAB2-MKK4-JNK signaling among MAPKs. One of candidates that exert the LECT2-mediated specificity in signaling pathway may be a scaffold protein. Previously, it was reported that scaffold protein JIP1 (JNK interaction protein 1) could bind to JNK and not to other MAPK such as p38 and ERK and specifically modify the MAPK signaling pathway^29^. It might be possible that LECT2 interferes with JIP1 and thereby specify the JNK phosphorylation under the LPS stimulation, the hypothesis that should be tested in future.

In conclusion, the present study demonstrates that hepatic expression of LECT2 is upregulated in association with the inflammatory signature in human liver tissues. The overnutrition-induced elevation of LECT2 activates the LPS-induced TAB2-MKK4-JNK axis as an adjuvant, shifts liver residual macrophages to the M1-like phenotype, and contributes to the development of liver inflammation. These findings shed light on the previously unrecognized function of the hepatokine LECT2 that may be a potential therapeutic target to dissociate liver steatosis from inflammatory signaling.

## Patients and methods

### Human clinical studies

Liver samples to be analyzed were obtained from 32 patients who underwent liver biopsy for the evaluation of NAFLD. Detailed clinical information about these subjects is presented elsewhere (Table 1).

This study was approved by the regional ethics committee (Medical Ethics Committee of Kanazawa University, No. 194111) All study procedures were in accordance with the Helsinki Declaration, and all study participants provided written informed consent.

### Animals and experimental design

C57BL/6J mice (8 week old) were obtained from Sankyo Laboratory Service (Tokyo, Japan). All animals were housed in a 12-h light/12-h dark cycle and allowed free access to food and water. A 60% high-fat diet (HFD; D03062301) was purchased from Research Diets (New Brunswick, NJ).

All animal procedures were in accordance with the standards set forth in the Guidelines for the Care and Use of Laboratory Animals at the Takara-machi campus of Kanazawa University, Japan.

### *Lect2* knockout mice

*Lect2* knockout (KO) mice were produced by homologous recombination using genomic DNA cloned from an Sv-129 P1 library, as described previously^30^. All experimental mice were generated from an intercross between heterozygous mice.

### Blood samples assays in mice

Serum levels of LECT2 were measured by the Ab-Match ASSEMBLY Mouse LECT2 kit (MBL International, Woburn, MA, USA). Fasting serum insulin levels were determined using a mouse insulin ELISA kit (Morinaga Institute of Biological Science, Inc., Yokohama, Japan), according to the manufacturers’ instructions.

### Measurement of hepatic triglyceride content in mice

After liver tissue weight was measured, frozen liver tissue was homogenized in 2 mL ice-cold isopropanol. The samples were incubated for 10 min with shaking at room temperature, and the samples were centrifuged at 3000 rpm for 10 min. 1 mL of supernatant was used to measure the hepatic triglyceride content by commercial Triglyceride E-test WAKO kit (Wako Pure Chemical Industries, Osaka, Japan). Results were normalized to the weight of each liver samples.

### RNA isolation, cDNA synthesis, and real-time PCR analysis

Total RNA was extracted from mouse liver by RNeasy Tissue Mini Kit (Qiagen). Reverse transcription of total RNA was performed using a high-capacity cDNA archive kit (Applied Biosystems, Foster City, CA). Real-time PCR analysis was performed by TaqMan gene expression assays (Applied Biosystems). Primer sets and TaqMan probes were proprietary to Applied Biosystems (Assays-on-Demand gene expression products).

### Preparation of recombinant murine LECT2

Murine LECT2 was prepared as previously described^31^. Briefly, murine LECT2 was expressed in Expi293 cells (Thermo Fisher Scientific, Waltham, MA, USA) in accordance with the manufacturer’s protocol. The cells were grown for 5–7 days after transfection, and the cell culture supernatant was diluted in 20 mmol/L phosphate buffer without sodium chloride, and potassium chloride, pH 7.2 (buffer A) and loaded onto a HiTrap CM FF column (GE Healthcare Bioscience, Piscataway, NJ, USA) equilibrated with buffer A. After the column was washed with buffer A, the protein was eluted with a linear gradient of 0–1 mol/L sodium chloride. The purified LECT2 was concentrated using VivaSpin (GE Healthcare). To determine the protein concentration, absorbance at 280 nm was measured.

### Treatment with recombinant LECT2 protein in KUP5

Studies were peformed using KUP5, mouse kupffer cell line^16^ (RCB4627, RIKEN BRC). Cells were cultured in 12-well multi plates, in high-glucose DMEM media with 10% fetal bovine serum (FBS), 10 μg/ml bovine insulin, and 250 × 10–6 m 1-thioglycerol. Cells were treated with recombinant LECT2 protein for 30 min with or without LPS.

### Western blot studies in KUP5

Western blot studies were performed as previously described^6^. After the LPS and LECT2 stimulation, treated cells were collected and lysed in 1 × RIPA lysis buffer (Upstate Biotechnology) with a Complete Mini EDTA-free cocktail tablet (Roche Diagnostics) and PhosSTOP phosphatase inhibitor cocktail tablets (Roche Diagnostics). Protein samples were subjected to SDS-PAGE and transferred to polyvinylidene fluoride membranes, using an iBlot gel transfer system (Invitrogen). Membranes were blocked and then they were probed with antibodies for 16 h (Phospho-SAPK/JNK (Thr183/Tyr185) Antibody (Cell Signaling, Danvers, MA), SAPK/JNK Antibody (Cell Signaling, Danvers, MA), Phospho-p38 MAPK (Thr180/Tyr182) Antibody (Cell Signaling, Danvers, MA), p38 MAPK Antibody (Cell Signaling, Danvers, MA), Phospho-p44/42 MAPK(Erk1/2) (Thr202/Tyr204) Antibody (Cell Signaling, Danvers, MA), p44/42 MAPK(Erk1/2) Antibody (Cell Signaling, Danvers, MA) Phospho-SEK1/MKK4 (Ser257) Antibody (Cell Signaling, Danvers, MA), SEK1/MKK4 Antibody (Cell Signaling, Danvers, MA) Afterward, membranes were washed and then incubated with anti-rabbit IgG horseradish peroxidase-linked antibody (Cell Signaling) for 1 h. Protein bands were visualized with ECL Prime Western blotting detection reagent (GE Healthcare UK Ltd.).

### Flow cytometry

Liver tissues from male C57BL/6J mice were minced and digested for 30 min at 37 °C with type II collagenase (Sigma-Aldrich) in PBS containing 2% BSA (pH 7.4). The cell suspension was filtered and then spun at 3000 rpm for 5 min to separate the floating liver tissue fraction from the stromal vascular cell (SVC) pellet. The SVCs were resuspended in PBS supplemented with 2% FBS and incubated with Fc-Block (BD Bioscience).

To determine macrophage phenotype fluorescence-activated cell sorting analysis FACSAriaII (BD Bioscience) was performed using following antibodies, NK1.1-PerCP Cy5.5 (eBioscience, San Diego, CA), CD3-PerCP Cy5.5 (eBioscience, San Diego, CA), CD19-PerCP Cy5.5(eBioscience), TER119-PerCP Cy5.5 (eBioscience, San Diego, CA), CD45-APC-Cy7(eBioscience, San Diego, CA), Gr-1Fluor450 Ly-6G(PB) (eBioscience, San Diego, CA), F4/80-PE-Cy7(Bio Legend, San Diego, CA), CD11b-PETR (Invitrogen, Carlsbad, CA, USA), CD11c-PE(eBioscience, San Diego, CA), CD206 Alexa Fluor 647 (Bio Legend, San Diego, CA). Data analysis and compensation were performed using FlowJo (Tree Star).

### Statistical analysis

All data were analyzed using the Japanese Windows Edition of SPSS version 21.0. Numeric values are reported as the mean ± SEM. Differences between the two groups were assessed using unpaired two-tailed t-tests. Data involving more than two groups were assessed by ANOVA.

## Supplementary Information

Supplementary Information.

## Abbreviations

NAFLD: Nonalcoholic fatty liver disease
NASH: Nonalcoholic steatohepatitis
LECT2: Leukocyte cell-derived chemotaxin 2
AST: Aspartate transferase
ALT: Alanine transaminase
HbA1c: Hemoglobin A1c
AMPK: Adenosine monophosphate-activated protein kinase
JNK: C-Jun N-terminal kinase
ERK: Extracellular signal-regulated kinases
LPS: Lipopolysaccharide
MAPK: Mitogen-activated protein kinases
