# Supplementary material for: LECT2 as a hepatokine links liver steatosis to inflammation via activating tissue macrophages in NASH
Source: Sci Rep. 2021 Jan 12;11:555. doi: 10.1038/s41598-020-80689-0 (PMC7804418; doi:10.1038/s41598-020-80689-0)

LECT2 as a hepatokine links liver steatosis to inflammation via activating tissue macrophages in NASH

Noboru Takata[1, 2], Kiyo-aki Ishii[7], Hiroaki Takayama[1, 6], Mayumi Nagashimada[3], Kyoko Kamoshita[1], Takeo Tanaka[1] Akihiro Kikuchi[1], Yumie Takeshita[1], Yukako Matsumoto[1], Tsuguhito Ota[1], Yasuhiko Yamamoto[4], Satoshi Yamagoe[5], Akihiro Seki[2], Yoshio Sakai[2], Shuichi Kaneko[2], Toshinari Takamura[1]

[1] Department of Endocrinology and Metabolism, Kanazawa University Graduate School of Medical Sciences, Kanazawa, Ishikawa 920-8640, Japan

[2] Department of Gastroenterology, Kanazawa University Graduate School of Medical Sciences, Kanazawa, Ishikawa 920-8640, Japan

[3] Technology Department of Clinical Laboratory Science, Kanazawa University Graduate school of Medical Science and Technology, Kanazawa, Ishikawa 920-0942, Japan

[4] Department of Biochemistry and Molecular Vascular Biology, Kanazawa University Graduate School of Medical Science, Kanazawa, Ishikawa 920-8640, Japan

[5] Department of Chemotherapy and Mycoses, National Institute of Infectious Diseases, Shinjuku-ku, Tokyo 162-8640, Japan

[6] Life Sciences Division, Engineering and Technology Department, Kanazawa University, Kanazawa, Ishikawa 920-8640, Japan

[7] Department of Integrative Medicine for Longevity, Kanazawa University Graduate

School of Medical Sciences, Kanazawa, Ishikawa 920-8640, Japan

Keywords: c-Jun N-terminal kinase, lipopolysaccharide, liver inflammation, nonalcoholic fatty liver disease, obesity,

List of abbreviations: NAFLD, nonalcoholic fatty liver disease; NASH, nonalcoholic steatohepatitis; LECT2, Leukocyte cell-derived chemotaxin 2; M, male; F, female; ALT, alanine transaminase; AMPK, adenosine monophosphate-activated protein kinase

Corresponding author: Toshinari Takamura, MD, PhD
Email: ttakamura@med.kanazawa-u.ac.jp
ORCID ID: https://orcid.org/0000-0002-4393-3244
Department of Endocrinology and Metabolism, Kanazawa University Graduate School of Medical Sciences, Kanazawa, Japan, 920-8640, Japan
Tel: 81-76-265-2711; Fax: 81-76-234-4214

Supplementary Information

Supplementary Figure S1

Supplementary Fig. S1 (A) Western blot analysis of JNK, ERK and p38 phosphorylation in KUP5. The cells were treated with LPS and LECT2 protein for 30 minutes with or without polymyxin B. (B) Western blot analysis of MKK4 and TAB2 phosphorylation in KUP5. The cells were treated with LPS and LECT2 protein for 30 minutes with or without polymyxin B.

Supplementary Fig. 1

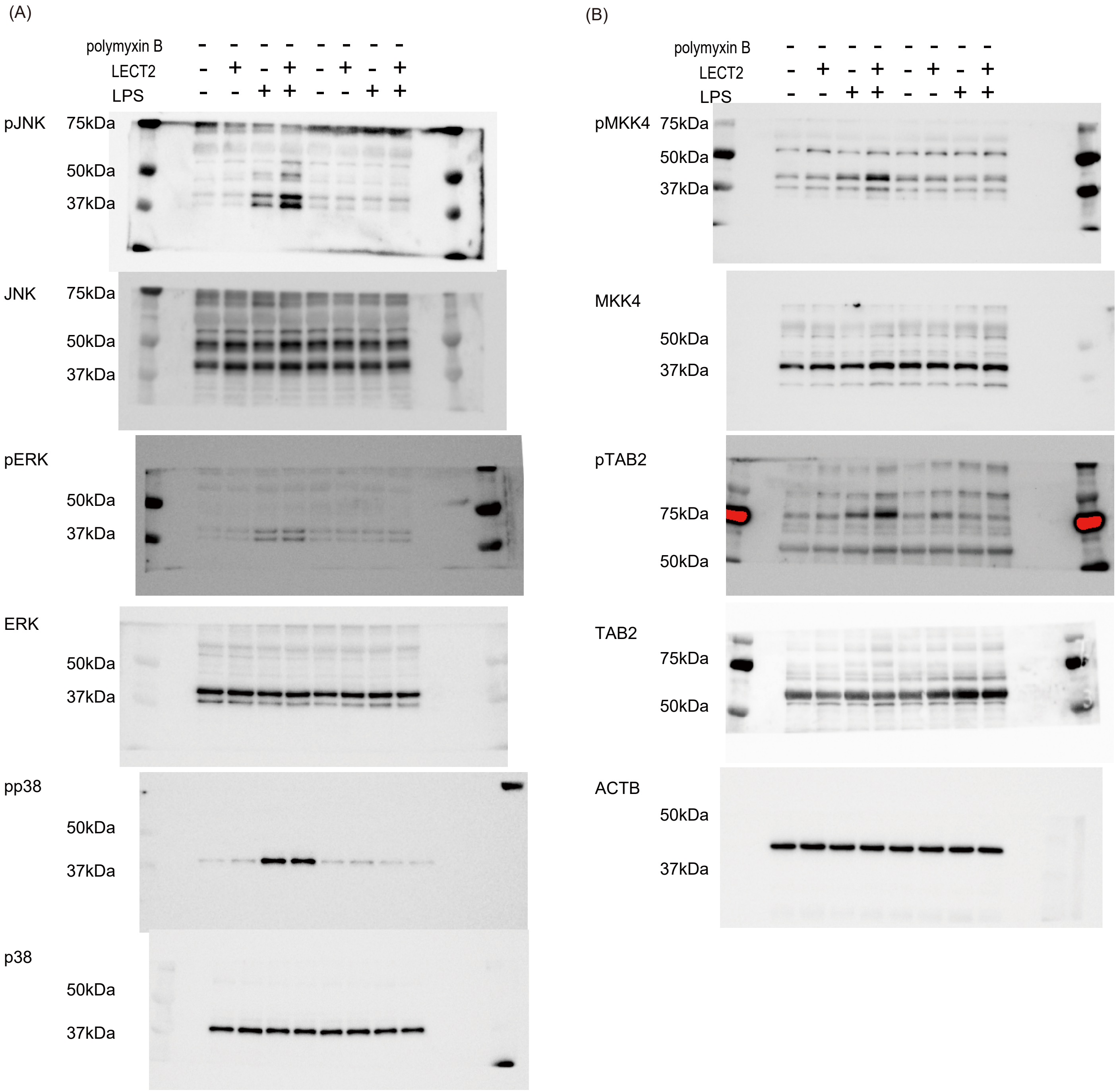

Supplement: Supplementary file 1 — Supplementary Information. [file 41598_2020_80689_MOESM1_ESM.pdf]
